# Frequency multiplexing for quasi-deterministic heralded single-photon sources

**DOI:** 10.1038/s41467-018-03254-4

**Published:** 2018-02-27

**Authors:** Chaitali Joshi, Alessandro Farsi, Stéphane Clemmen, Sven Ramelow, Alexander L. Gaeta

**Affiliations:** 1 Department of Applied Physics and Applied Math, Columbia University, New York, NY 10027 USA; 2 Applied and Engineering Physics, Cornell University, Ithaca, NY 14850 USA; 3 Laboratoire d’ Information Quantique, Université Libre de Bruxelles, Bruxelles, 1050 Belgium; 4 Institut für Physik, Humboldt-Universität zu Berlin, Berlin, 12489 Germany

## Abstract

Parametric single-photon sources are well suited for large-scale quantum networks due to their potential for photonic integration. Active multiplexing of photons can overcome the intrinsically probabilistic nature of these sources, resulting in near-deterministic operation. However, previous implementations using spatial and temporal multiplexing scale unfavorably due to rapidly increasing switching losses. Here, we break this limitation via frequency multiplexing in which switching losses remain fixed irrespective of the number of multiplexed modes. We use low-noise optical frequency conversion for efficient frequency switching and demonstrate multiplexing of three modes. We achieve a generation rate of 4.6 × 10^4^ photons per second with an ultra-low *g*^(2)^(0) = 0.07 indicating high single-photon purity. Our scalable, all-fiber multiplexing system has a total loss of just 1.3 dB, such that the 4.8 dB multiplexing enhancement markedly overcomes switching loss. Our approach offers a promising path to creating a deterministic photon source on an integrated chip-based platform.

## Abstract (web summary)

The aim of multiplexing is to boost capabilities of probabilistic single photon sources, but is vexed by rapidly increasing switching losses. Here, the authors propose and implement an in-fiber frequency-multiplexing scheme where total losses are independent of the number of multiplexed modes.

## Introduction

Deterministic and high quality single-photon sources are essential to photonic quantum technologies including communications and information processing. An ideal single-photon source should emit indistinguishable photons in well-defined spatio-temporal and spectral modes with high probability and negligible multi-photon noise. Efforts to build such sources have focused primarily on the following two approaches: sources that rely on nonlinear processes such as spontaneous parametric down conversion (SPDC) or four-wave mixing, and single emitters such as quantum dots, color centers, and cavity-coupled atoms and ions^1^. With recent engineering efforts for improved fabrication and control of individual emitters, quantum dots with high brightness and photon purity have been demonstrated^2–4^.

Parametric sources have specific advantages such as spectral tunability and can be easily adapted to a wide variety of experimental conditions. They have thus been used for pioneering quantum information experiments including quantum teleportation, loophole-free Bell tests, and boson sampling^5–9^. These sources operate at room temperature and provide highly indistinguishable photons with flexible control over the spectral and temporal properties of the photons^10–13^. Such sources have proved to be highly versatile, producing photons spanning the visible to the infrared, with bandwidths ranging from a few hundred kHz to a few THz^14–16^. Moreover, parametric sources can be fully integrated onto monolithic CMOS-compatible platforms to generate narrow band entangled photons with high brightness^17–19^. However, these sources are fundamentally limited by multi-photon generation, resulting in probabilistic operation with a maximum heralding efficiency of 25% from a single source.

Active feed-forward switching of photons from multiple identical sources is a promising technique that can overcome the probabilistic operation of a single source^20–24^. By operating individual sources in a regime with low pair production probability, such schemes allow for increasing the single-photon probability without additional multi-photon generation. A key requirement for efficient multiplexing is a low-loss *N* × 1 switching network that accommodates a sufficiently large number of modes *N* to achieve deterministic operation. Deterministic operation can be achieved with as few as *N* = 17 multiplexed modes with a lossless switching network and photon-number resolving (PNR) detectors^25^. Recently, there have been a number of promising demonstrations of multiplexed sources using the spatial and temporal degrees of freedom of a photon^26–32^. However, for both spatial and temporal multiplexing, switching losses increase with the number of modes *N*, which deteriorates enhancement achieved from multiplexing beyond a few modes. Deterministic operation is therefore challenging to achieve without the use of bulky free-space setups^31^.

Here, we propose and demonstrate an alternative scheme using frequency multiplexing where losses do not scale with the number of modes. Frequency multiplexing allows for multiple switching operations in a single spatial mode, thus effectively implementing an *N* × 1 switch in a monolithic optical structure such as a single mode fiber or waveguide. Therefore, distinct from other schemes, switching losses remain fixed irrespective of the number of multiplexed modes *N*. We perform active “frequency switching” using tunable quantum frequency translation via Bragg scattering four-wave mixing (BS-FWM)^33–36^, which we realize with close to unity efficiency and ultra-low noise^37,38^. We present a proof-of-principle demonstration of frequency multiplexing using three frequency modes in an entirely fiber-based setup that leverages on low-loss off-the-shelf dense wavelength division multiplexing (DWDM) components. With this low-loss and low-noise setup we achieve generation rates of 46 kHz multiplexed photons with coincidences-to-accidentals ratio exceeding 100 and *g*^(2)^(0) of 0.07. In addition, we show that BS-FWM is efficiently tunable over a large bandwidth of more than 1 THz and therefore our system can be scaled to include a large number of frequency modes, which is critical for deterministic photon generation using multiplexing.

## Results

### Principle and theory

**Fig. 1:**
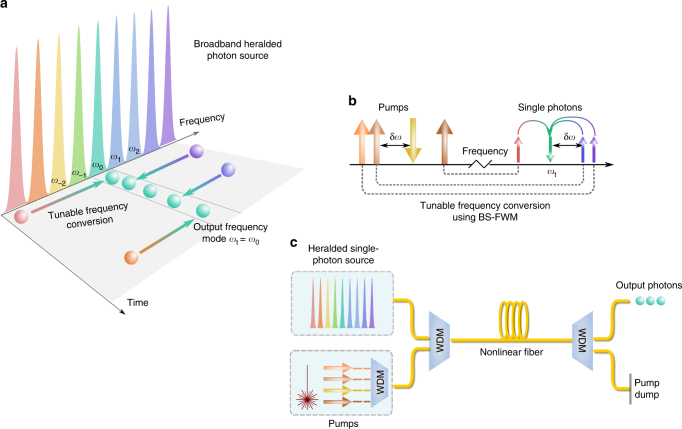
Principle of a frequency multiplexed single-photon source. **a** Multiple narrowband frequency channels {*ω*_0_, *ω*_1_...} are extracted from a broadband single-photon source. Tunable frequency conversion is used to convert photons from different channels to a common target frequency mode *ω*_t_. **b** Tunable frequency conversion using Bragg scattering four-wave mixing (BS-FWM): two strong classical pumps drive the interaction between the input and target (*ω*_t_) single-photon fields. The frequency separation δ*ω* between the pump fields determines the frequency shift of the single photons, and additional pump fields can be used to increase the number of possible values of δ*ω*. **c** Fixed-loss operation of frequency multiplexing: single photons and BS-FWM pumps are combined using wavelength division multiplexers (WDM), and all active frequency switching takes place in a single nonlinear fiber/waveguide. Additional channels can be added by introducing additional pumps, without introducing losses in the path of the single photons

Figure 1 illustrates our frequency multiplexing scheme. A single source that generates broadband frequency correlated photon pairs is used to create narrowband frequency channels {*ω*_0_, *ω*_1_...}. One photon from the pair (heralding photon, not shown) is used to herald the presence of the signal photon. Due to energy conservation, the two photons are correlated in frequency, with the heralding photon providing information about the frequency of the signal photon. This heralding information is used to translate the frequency of the signal photon to the target frequency channel *ω*_t_ using tunable frequency conversion. We thus effectively implement an active frequency switch to route photons from multiple frequency bins to a single output frequency channel. In order to be viable as an *N* × 1 switch for large *N*, the tunable frequency conversion must be efficient over a sufficiently large bandwidth. For this purpose, we use BS-FWM, a third-order nonlinear parametric process involving coherent interaction between two quantum fields at different frequencies mediated by two strong classical pumps^35^. Contrary to frequency conversion based on parametric amplification, BS-FWM is theoretically noiseless and preserves all quantum properties of the translated photons. BS-FWM allows for independent control of the input and target frequencies by selectively activating auxiliary pumps in the interaction (Fig. 1b). Since phase matching can be achieved by symmetric placement of the classical pumps and quantum fields about the zero-dispersion wavelength of the nonlinear medium, the same setup can be reconfigured to target different frequency shifts by tuning the pump wavelength (Supplementary Note 1). For efficient conversion, it is critical that the bandwidth of individual channels be less than the acceptance bandwidth Δ*ν*_BS_ of the BS-FWM process for two fixed pumps. This all-optical frequency switch can support ultrafast operation, with the repetition rate limited only to the inverse bandwidth 1/Δ*ν*_BS_. Finally, we note that all frequency switching takes place in a single spatial mode (nonlinear fiber/waveguide), as shown in Fig. 1c. As additional channels only require additional BS-FWM pumps, no scaling losses are introduced in the path of the single photons.

To understand clearly the characteristics of our frequency multiplexing scheme, we analyze how the performance scales with increasing *N*. Several architectures have been explored for active *N* × 1 switching of photons in spatial and temporal multiplexing schemes. Typically, these architectures use 2 × 2 switches as building blocks for a general *N* × 1 switch. We compare the performance of the fixed-loss scheme with the log-tree network which is generally used for spatial multiplexing and multi-pass binary switches (or storage cavities) generally used in temporal multiplexing^30,31^. An *N* × 1 log-tree network has a depth $\lceil\log_{2} N \rceil$. Assuming a switching efficiency of *η*_s_ per switch, the losses scale as $\eta_{s}^{\lceil \log_{2}N \rceil}$^39^. The losses from multi-pass binary switching scale exponentially as $\eta _{\rm{s}}^N$ in the worst case, but we consider an optimized implementation as in ref. ^31^. For our fixed-loss scheme, the switching losses are *η*_s_ irrespective of *N*.

**Fig. 2:**
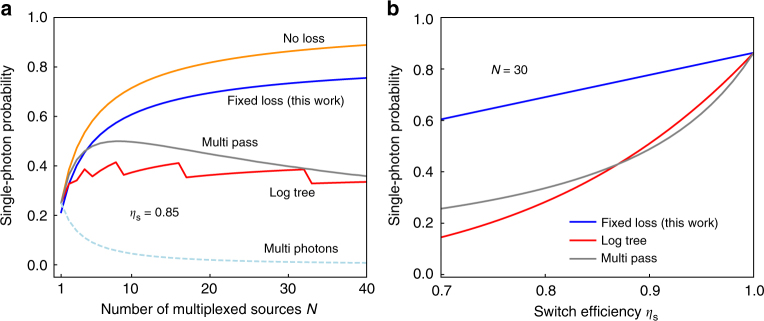
Theoretical prediction of scaling performance for various switching schemes. **a** The maximum single-photon heralding probability for a single source (*N* = 1) is 0.25. For an efficiency of *η*_s_ = 0.85 (0.7 dB loss) per switch, the single-photon emission probability for both log-tree and multi-pass schemes reaches a maximum of 0.41, 0.50, respectively, and then saturates for large *N* due to scaling losses. In contrast, additional multiplexed sources always result in improved performance for the fixed-loss scheme, with *p*_mux_(*n* = 1) = 0.75 for *N* = 40 sources. The multi-photon emission probability *p*_mux_(*n* > 1), ignoring switching losses, is shown as the dashed light blue curve, and is <1% for *N* = 40 modes. **b** Single-photon heralding probability for various switching schemes as a function of switch loss, for a fixed number of sources *N* = 30. The fixed-loss scheme is significantly more robust to variability in switching losses. Note that the maximum heralding probability for *η*_s_ = 1 is not equal to 1 since the heralding detectors are non-photon-number resolving

Figure 2a shows the scaling performance of various schemes. We assume a switching efficiency *η*_s_ = 0.85 (0.7 dB loss) per switch and all other components, including detectors, are assumed to be ideal. We optimize the emission probability per source *p*_single_(*n* = 1) for each *N* (Supplementary Note 2). The maximum heralding probability for a single source (*N* = 1) is 0.25. For both log-tree and multi-pass schemes, the single photon probability reaches a maximum of 0.41 and 0.50, respectively, and saturates due to switching losses for less than *N* = 10 multiplexed sources. In contrast, for the fixed-loss scheme, additional multiplexed sources always result in an improvement in the single-photon heralding probability, with a heralding probability of 0.60 for *N* = 10 sources. For *N* = 40 sources, the fixed-loss scheme achieves *p*_mux_(*n* = 1) = 0.75, compared with a maximum of 0.89 with a no-loss ideal switching network. Our scheme therefore has an advantage in the intermediate regime of 10 to 20 multiplexed modes as well as asymptotically for large *N*. In order to quantify the effects of practical variability in switching efficiency in implementations of multiplexed sources, we analyze the sensitivity of different schemes to switching losses in Fig. 2b. For a moderate increase in losses to 1.2 dB per switch and 30 multiplexed modes (*η*_s_ = 0.75, *N* = 30), the single-photon probability drops significantly from 0.86 (*η*_s_ = 1) to 0.21, 0.29 for the log-tree and multi-pass schemes, but is reduced only moderately to 0.65 for the fixed-loss scheme. Thus, the frequency multiplexing scheme is significantly more robust to switching losses as compared to competing switching architectures in other multiplexed sources.

### Experimental setup

**Fig. 3:**
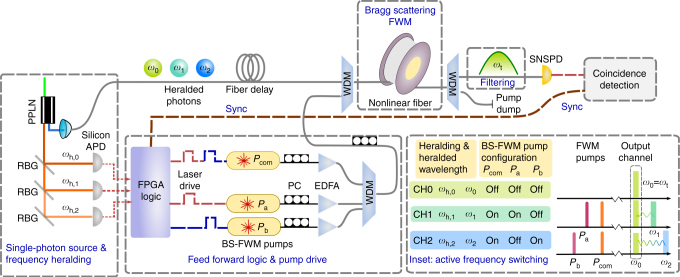
Experimental setup for multiplexing of three frequency modes. PPLN periodically poled lithium niobate, RBG reflecting Bragg grating, FPGA field programmable gate array, PC polarization control, EDFA erbium-doped fiber amplifier, WDM wavelength division multiplexer, BS-FWM Bragg scattering four-wave mixing, SNSPD superconducting nanowire single-photon detector, APD avalanche single-photon detector. A PPLN crystal is pumped with a CW laser at 543 nm, generating photon pairs at 940 nm (heralding photons) and 1280 nm (heralded photons). The heralding photons are filtered into three channels {*ω*_h,0_, *ω*_h,1_, *ω*_h,2_} with 100 GHz bandwidth, with corresponding heralded signal photons as {*ω*_0_, *ω*_1_, *ω*_2_}. An FPGA is used to process this heralding information and selectively activate the BS-FWM pumps (see inset), such that the heralded signal photons are switched to the target frequency *ω*_t_. The signal photons are combined with the BS-FWM pumps using WDMs, and sent to the nonlinear fiber. A free-space filtering setup extracts photons at *ω*_t_ at the output, which are then sent to an SNSPD. A time-tagging module is used for coincidence measurements between the FPGA processed heralding trigger (sync) and the output of the SNSPD

We experimentally demonstrate multiplexing of three frequency modes. Figure 3 shows our experimental setup. Our multiplexed source is based on broadband SPDC in a periodically poled lithium-niobate crystal (PPLN) pumped with a 543-nm CW-laser, generating photon pairs at 940 nm (heralding photons) and 1280 nm (heralded signal photons). The heralding photons are sent to a filtering setup consisting of reflecting Bragg gratings (RBG), creating three channels CH0, CH1, CH2 with heralding photons at *ω*_h,0_, *ω*_h,1_, *ω*_h,2_, respectively, with 100-GHz bandwidth (Supplementary Note 3). Each channel is collected into a single-mode fiber and sent to a silicon avalanche single-photon detector (APD), which provides heralding information to the logic circuit. The source crystal temperature is tuned to maximize photon pair production at *ω*_1_ = 1280.65 nm and *ω*_2_ = 1280.1 nm, and the pair production at *ω*_0_ = 1284.45 nm is lower by a factor of 0.65. The heralded signal photons {*ω*_0_ = 1284.45 nm, *ω*_1_ = 1280.65 nm, *ω*_2_ = 1280.1 nm} are injected into the multiplexing setup, comprised of a 100-m nonlinear fiber, wavelength-division-multiplexing couplers and a pump filter. A single channel centered at *ω*_t_ = 1284.45 nm and 100 GHz wide, is selected with a tunable grating and then sent to a superconducting nanowire single-photon detector (SNSPD) with a quantum efficiency of 53%.

The nonlinear process of BS-FWM is driven by two pump waves generated by distributed feedback lasers diodes, which determine the frequency shift and hence the input and output frequency channels. The diodes are driven with a 5-ns-long pulsed current source, and the optical pulses (for convenience aligned to the C-band ITU grid) are amplified to a peak level of 10 W via cascaded erbium-doped fiber amplifiers (EDFA). The pump pulses are combined together, temporally synchronized and aligned in polarization. In order to achieve fast switching operations, we utilize lasers at predetermined wavelengths that are selectively turned on and off via a fast logic circuit controlled by a field programmable gate array (FPGA) (see inset in Fig. 3). We measure the conversion efficiency for both process *ω*_1_ → *ω*_t_ and *ω*_2_ → *ω*_t_ to be 93% (Supplementary Note 1).

### Single-photon rate

**Fig. 4:**
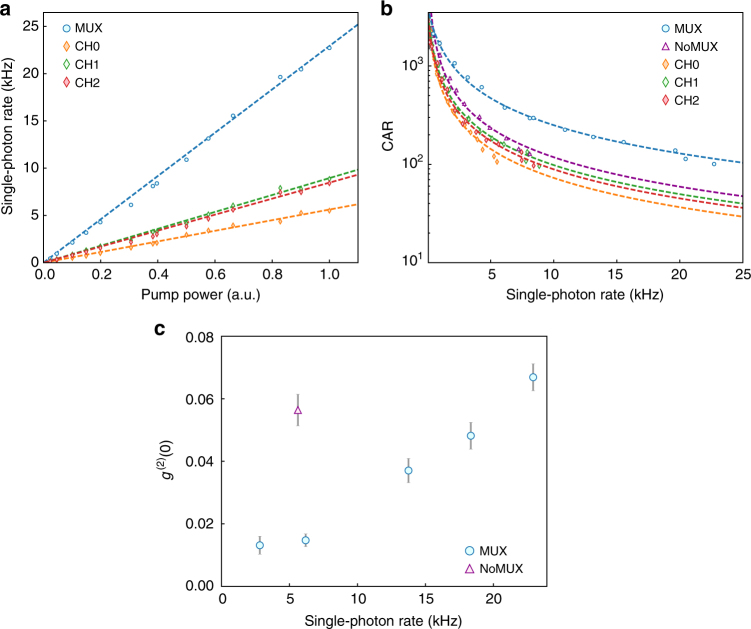
Experimental results from multiplexing of three frequency modes. Blue circles: multiplexed (MUX) source, purple triangles: NoMUX source, diamonds: contributions from the individual channels (orange—CH0, green—CH1, red—CH2). **a** Single-photon rate (coincidence counts) as functions of SPDC pump power: The multiplexed source has an enhanced single-photon rate by 4.8 dB as compared to the mean of the individual channels and overcomes the losses of the setup (1.3 dB), with a net enhancement of 220%. At maximum SPDC pump power (25 mW), we measure a single-photon rate of 23 kHz from the multiplexed source. **b** Coincidences-to-accidentals ratio (CAR) vs single-photon rate: For a fixed single-photon rate, the multiplexed source has a CAR that is a factor of 2 higher as compared to the NoMUX source. For low count rates, the multiplexed source has a CAR exceeding 1000 and remains high at 100 for large single-photon rates. **c** Measurement of *g*^(2)^(0): the multiplexed source has a low *g*^(2)^(0) of 0.07 ± 0.005 for large single-photon rates. For the same single-photon rate of 5.6 kHz the multiplexed source has an improved single-photon purity with *g*^(2)^(0) of 0.015 ± 0.002 as compared to the NoMUX source with a *g*^(2)^(0) of 0.056 ± 0.005. Error bars are estimated using Poisson statistics

We first characterize the single-photon rates as functions of SPDC pump power for each individual channel and for the multiplexed source, as shown in Fig. 4a. The multiplexed (MUX) source has an enhanced coincidence rate by 4.8 dB as compared to the mean photon rate of the individual channels. This enhancement significantly overcomes the losses of the setup (1.3 dB), resulting in a net enhancement of 3.5 dB (220%) in the single-photon rate. At maximum SPDC pump power (25 mW), we measure a heralding rate of 1 MHz with a brightness of 23 kHz detected coincidences per second. We estimate a single-photon generation rate of 46 kHz after correcting for detector efficiency (3 dB). Supplementary Note 4 provides detailed characterization of the system efficiency and losses. We note that although simply increasing the pump power of the SPDC source can increase the single-photon generation rate of a single source, this would lead to increased multi-photon generation.

### Coincidences-to-accidentals ratio

We measure the coincidences-to-accidentals ratio (CAR), a standard figure of merit to characterize the multi-photon generation of parametric sources. Figure 4b compares the CAR for the multiplexed source and each individual channel. For fair comparison we also measure the coincidence rate and CAR at *ω*_t_, directly from the SPDC source, without the multiplexing setup in place (referred to as the NoMUX source). We operate in a regime in which the single-photon count rate is much higher than the dark-count rate of the detectors, and therefore the accidental counts are dominated by multi-photon generation, which is inversely proportional to the SPDC pump power. The multiplexed source has a CAR that is a factor of 2 higher throughout as compared to the NoMUX source. For low count rates, the multiplexed source has a CAR exceeding 1000 and remains high at 100 at the maximum count rate. These measurements confirm that the strong classical pumps used in BS-FWM do not introduce significant spurious noise photons even at a high pump trigger rate of 1 MHz.

### Single-photon purity

Finally, we measure the purity of the photons from the multiplexed source by the second-order correlation function $g^{(2)} = \left\langle {\hat N_{\mathrm{a}}\hat N_{\mathrm{b}}} \right\rangle /\left\langle {\hat N_{\mathrm{a}}} \right\rangle \left\langle {\hat N_{\mathrm{b}}} \right\rangle$ where $\hat N_{\mathrm{a}}$ and $\hat N_{\mathrm{b}}$ are photon number operators corresponding to the two arms of a Hanbury-Brown–Twiss setup^40^. Figure 4c shows the measured *g*^(2)^(0) for the multiplexed source and the NoMUX source, for various single-photon rates. At the maximum count rate, the multiplexed source has a low *g*^(2)^(0) of 0.07 ± 0.005. For the same low single-photon rate of 5.6 kHz, the multiplexed source and the NoMUX source have *g*^(2)^(0) of 0.015 ± 0.002 and 0.056 ± 0.005 respectively. The average SPDC pump power required to achieve the same photon rate is a factor of 3 lower for the multiplexed source as compared to the NoMUX source, and therefore has significantly reduced multi-photon generation. The improved single photon purity of the multiplexed source is therefore a strong indicator of successful multiplexing.

The performance of our frequency multiplexed source is comparable with the best multiplexed source demonstrated to-date which implements temporal multiplexing on a free-space optics platform^31^. A complete comparison with other relevant works has been included in Supplementary Note 5. We achieve a single-photon generation rate of 46 kHz with an ultra-low *g*^(2)^(0) of 0.07, compared with previously demonstrated 19.3 kHz with a high *g*^(2)^(0) of 0.48^31^. Due to the low loss of our frequency switch (1.3 dB), we achieve a multiplexing enhancement factor of 2.2 with just three frequency modes. We measure a raw heralding efficiency of 2.3% and detector-corrected efficiency of 4.6%. This efficiency is mainly limited by the fiber-collection and spectral filtering loss at the SPDC source and is independent of our “frequency switching” setup. Collection efficiencies as high as 90% can be achieved by minimizing all transmission and filtering losses, and careful mode-matching, which would correspond to an order of magnitude improvement in the single-photon generation rates^9,41^. Another important figure of merit for comparing the different multiplexing implementations is the maximum possible switching speed. In principle, our all-optical frequency switch allows for efficient conversion with repetition rates as high as the inverse of the BS-FWM acceptance bandwidth (100 GHz in this system). Our current implementation can support a repetition rate of 5 MHz and is only limited by the amplification required for the BS-FWM pumps. This amplification requirement can be reduced by increasing the BS-FWM interaction length or using highly nonlinear fibers as the interaction medium, enabling significantly higher repetition rates.

### Pulsed operation and scaling

**Fig. 5:**
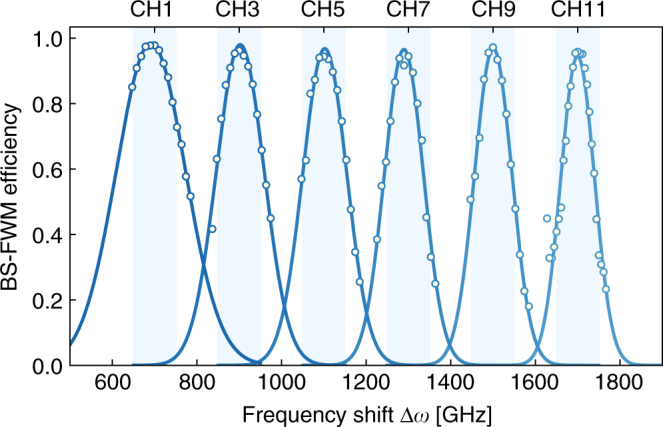
Bragg scattering four-wave mixing (BS-FWM) scaling: measured BS-FWM efficiency for frequency shifts Δ*ω* between the input and target frequency varying from 700 GHz (CH1) to 1700 GHz (CH11). One pump in the BS-FWM interaction is tuned in steps of 200 GHz to increase the frequency shift between the input and target frequency. The other pump is detuned by a small amount (<10 GHz) such that phase matching remains optimal at the target frequency *ω*_t_ at 1284.45 nm. The frequency conversion efficiency is maintained at 95%, while the acceptance bandwidth is reduced by a factor of 2 due to effects of higher order dispersion. Assuming a 100 GHz channel bandwidth, this corresponds to efficient conversion over ten frequency modes

In order to approach deterministic photon generation, pulsed operation together with a large number of multiplexed modes is necessary. Our system supports pulsed operation at high repetition rates. The efficiency of our “frequency switch” is partially limited to 93% due to the fluctuations in BS-FWM pump power induced by the randomized trigger arising from CW operation of the single-photon source. We measure efficiencies as high as 95% using the same setup with periodic pump triggering. In Fig. 5 we show that BS-FWM remains efficient over ten frequency modes by tuning the frequency shift Δ*ω* between the input and target frequency from 700 GHz (CH1) to 1700 GHz (CH11). This is achieved by shifting one pump by over 1 THz in steps of 200 GHz and tuning the other pump by a small amount (<10 GHz) such that phase matching remains optimal at the target frequency *ω*_t_ (1284.45 nm). The frequency conversion efficiency is maintained at 95% while the acceptance bandwidth reduces by a factor of 2 due to effects of higher order dispersion (Supplementary Note 6). We summarize the predicted scaling performance of our system in Table 1. With just ten multiplexed modes, we expect that our system can achieve a single-photon heralding probability exceeding 50% (per input pump pulse) with a single-photon generation rate of 2.5 MHz (Supplementary Note 6).

**Table 1:** Performance of the frequency multiplexed source

	#Modes *N*	Multiplexing enhancement	Heralding efficiency	Single-photon generation rate	BS-FWM repetition rate
a	3	2.2	4.6%	46 kHz	1 MHz
b	10	8.5	50%	2.5 MHz	5 MHz

^a^ Current system with three modes and multiplexing system loss of 1.3 dB (*η*_s_ = 0.75)

^b^ Scaled source with ten modes and improved system efficiency (*η*_s_ = 0.85, fiber-collection and detection efficiency of 90%)

### Generation of indistinguishable photons

In addition to high brightness and purity, the generation of highly indistinguishable photons is critical to many quantum protocols. Due to CW operation and the highly non-degenerate nature of our downconversion source, a demonstration of indistinguishability through quantum interference of consecutive photons is beyond our current experimental setup. However, we have previously demonstrated Hong-Ou-Mandel interference between photons of different frequencies using BS-FWM as a frequency beamsplitter, indicating that the two photons are rendered indistinguishable after interaction^42^. Since our scheme uses narrow filtering, we can generate indistinguishable photons using a pulsed pump with the down-conversion phase-matching bandwidth matched to the filtering bandwidth. For example, using a short 1 mm PPLN crystal with 100 GHz channel filtering results in an expected indistinguishability of 89% (Supplementary Note 7). However, using a shorter crystal will inevitably reduce the brightness of the photon source. This tradeoff can be overcome using microresonator-based photon sources based on cavity-enhanced spontaneous four-wave mixing. By matching the spectral line-width of the pump pulse to the resonator line-width, it is possible to generate discrete uncorrelated joint spectral amplitudes with an expected indistinguishability of 92%^43^. Moreover, using advanced interferometric coupling, the effective pump resonance line-width can be broadened to generate fully unentangled photon pairs with an expected indistinguishability of more than 99%^44,45^. Therefore, using micro-resonator based sources together with frequency multiplexing, it is possible to generate indistinguishable photons in pure spectral and temporal modes.

## Discussion

We have demonstrated a frequency multiplexed source with three modes, using highly efficient low-noise quantum frequency translation. We emphasize that adding additional channels adds complexity only to the BS-FWM pump configuration, and no new components need to be added in the path of the single photons. This ensures that losses remain independent of the number of multiplexed modes. The single spatial mode operation of frequency switching maintains relative polarization stability of photons from different channels from generation to detection, ensuring that the photons are rendered indistinguishable after frequency translation. We note that recently, multiple research groups have proposed the use of frequency multiplexing as a resource for both continuous variable^46,47^ and circuit-based single-photon QIP applications^48,49^. These proposals emphasize the strong potential of frequency multiplexing for addressing the scaling losses and resource overheads in quantum systems. However, most proposals rely on electro-optic modulators (EOMs) to frequency translate single photons. Recent work^50^ discusses a spectrally multiplexed single-photon source using EOMs, but no enhancement in the single-photon rate is demonstrated due to high system losses. In addition, EOMs typically have a limited time-bandwidth product close to unity, limiting the maximum frequency shift and the bandwidth of the target pulses. This significantly limits practical implementations to a few frequency modes while exacerbating photon loss due to narrow filtering. Alternatively, our implementation of BS-FWM allows for tunable conversion over 1 THz with an acceptance bandwidth of 100 GHz with few nanosecond pump pulses, which addresses these issues. BS-FWM is fully compatible with the existing optical telecommunication architecture that harnesses DWDM. The applications of such low-loss high repetition rate frequency multiplexing go beyond single-photon sources and can prove to be highly advantageous for all-photonic quantum repeaters that rely on active feed-forward heralding signals^51^. Our scheme is also entirely adaptable to monolithic CMOS-compatible integrated platforms. In particular, integrated comb sources where photons are already confined in well-defined frequency bins can eliminate the need for filtering^17–19^ while generating spectrally pure photons^43^. Thermal tuning using integrated heaters can be used to precisely control and stabilize the microring resonance. Moreover, implementations of BS-FWM in nanophotonic waveguides can significantly reduce pump power and amplification requirements^52^ while dispersion engineering can provide flexible phase-matching conditions. The higher loss tolerance of frequency multiplexing makes it an ideal choice for integrated multiplexed sources as compared to other schemes which require free space optics to maintain low loss. The precision, flexibility and repeatability offered by integration will be critical for future quantum networks which will require thousands of identical photon sources operating simultaneously. Frequency multiplexing can thus uniquely harness both fiber and integrated technologies optimized for classical applications to address challenges of scalability in quantum technologies.

### Data availability

All data generated and/or analyzed during this study are available from the corresponding author on reasonable request.

## Electronic supplementary material

Supplementary Information

## References

[1] Eisaman MD, Fan J, Migdall A, Polyakov SV (2011). Invited review article: single-photon sources and detectors. Rev. Sci. Instrum..

[2] Loredo JC (2016). Scalable performance in solid-state single-photon sources. Optica.

[3] Ding X (2016). On-demand single photons with high extraction efficiency and near-unity indistinguishability from a resonantly driven quantum dot in a micropillar. Phys. Rev. Lett..

[4] Somaschi N (2016). Near-optimal single-photon sources in the solid state. Nat. Photonics.

[5] Ma XS (2012). Quantum teleportation over 143 kilometres using active feed-forward. Nature.

[6] Giustina M (2015). Significant-loophole-free test of Bell’s theorem with entangled photons. Phys. Rev. Lett..

[7] Broome MA (2013). Photonic Boson sampling in a tunable circuit. Science.

[8] Spring JB (2013). Boson sampling on a photonic chip. Science.

[9] Shalm LK (2015). Strong loophole-free test of local realism. Phys. Rev. Lett..

[10] Kwiat PG (1995). New high-intensity source of polarization-entangled photon pairs. Phys. Rev. Lett..

[11] Kwiat PG, Waks E, White AG, Appelbaum I, Eberhard PH (1999). Ultrabright source of polarization-entangled photons. Phys. Rev. A.

[12] Tanzilli S (2001). Highly efficient photon-pair source using periodically poled lithium niobate waveguide. Electron. Lett..

[13] Fedrizzi A, Herbst T, Poppe A, Jennewein T, Zeilinger A (2007). A wavelength-tunable fiber-coupled source of narrowband entangled photons. Opt. Express.

[14] Rambach M, Nikolova A, Weinhold TJ, White AG (2016). Sub-megahertz linewidth single photon source. APL Photonics.

[15] Nasr MB (2008). Ultrabroadband biphotons generated via chirped quasi-phase-matched optical parametric down-conversion. Phys. Rev. Lett..

[16] O’Donnell KA, U’Ren AB (2007). Observation of ultrabroadband, beamlike parametric downconversion. Opt. Lett..

[17] Reimer C (2014). Integrated frequency comb source of heralded single photons. Opt. Express.

[18] Reimer C (2016). Generation of multiphoton entangled quantum states by means of integrated frequency combs. Science.

[19] Ramelow, S. et al. Silicon-nitride platform for narrowband entangled photon generation. Preprint at https://arxiv.org/abs/1508.04358 (2015).

[20] Migdall AL, Branning D, Castelletto S (2002). Tailoring single-photon and multiphoton probabilities of a single-photon on-demand source. Phys. Rev. A.

[21] Jeffrey E, Peters NA, Kwiat PG (2004). Towards a periodic deterministic source of arbitrary single-photon states. New J. Phys..

[22] Pittman TB, Jacobs BC, Franson JD (2002). Single photons on pseudodemand from stored parametric down-conversion. Phys. Rev. A.

[23] Shapiro JH, Wong FN (2007). On-demand single-photon generation using a modular array of parametric downconverters with electro-optic polarization controls. Opt. Lett..

[24] Mower J, Englund D (2011). Efficient generation of single and entangled photons on a silicon photonic integrated chip. Phys. Rev. A.

[25] Christ A, Silberhorn C (2012). Limits on the deterministic creation of pure single-photon states using parametric down-conversion. Phys. Rev. A.

[26] Ma XS, Zotter S, Kofler J, Jennewein T, Zeilinger A (2011). Experimental generation of single photons via active multiplexing. Phys. Rev. A.

[27] Broome MA, Almeida MP, Fedrizzi A, White AG (2011). Reducing multi-photon rates in pulsed down-conversion by temporal multiplexing. Opt. Express.

[28] Collins MJ (2013). Integrated spatial multiplexing of heralded single-photon sources. Nat. Commun..

[29] Mendoza GJ (2016). Active temporal and spatial multiplexing of photons. Optica.

[30] Xiong C (2016). Active temporal multiplexing of indistinguishable heralded single photons. Nat. Commun..

[31] Kaneda F (2015). Time-multiplexed heralded single-photon source. Optica.

[32] Francis-Jones RJA, Hoggarth RA, Mosley PJ (2016). All-fiber multiplexed source of high-purity single photons. Optica.

[33] Inoue K (1994). Tunable and selective wavelength conversion using fiber four-wave mixing with two pump lights. IEEE Photonics Technol. Lett..

[34] McKinstrie CJ, Harvey JD, Radic S, Raymer MG (2005). Translation of quantum states by four-wave mixing in fibers. Opt. Express.

[35] McGuinness HJ, Raymer MG, McKinstrie CJ, Radic S (2010). Quantum frequency translation of single-photon states in a photonic crystal fiber. Phys. Rev. Lett..

[36] Li Q, Davanço M, Srinivasan K (2016). Efficient and low-noise single-photon-level frequency conversion interfaces using silicon nanophotonics. Nat. Photonics.

[37] Farsi, A., Clemmen, S., Ramelow, S. & Gaeta, A. L. Low-noise quantum frequency translation of single photons. In *Proc. Conference on Lasers and Electro-Optics*, FM3A.4 (Optical Society of America, 2015).

[38] Clemmen S, Farsi A, Ramelow S, Gaeta AL (2016). Ramsey interference with single photons. Phys. Rev. Lett..

[39] Bonneau D, Mendoza GJ, O’Brien JL, Thompson MG (2015). Effect of loss on multiplexed single-photon sources. New J. Phys..

[40] Brown RH, Twiss RQ (1956). Correlation between photons in two coherent beams of light. Nature.

[41] Ramelow S (2013). Highly efficient heralding of entangled single photons. Opt. Express.

[42] Joshi, C., Farsi, A. & Gaeta, A. L. Hong-Ou-Mandel Interference in the Frequency Domain. In *Proc. Conference on Lasers and Electro-Optics*, FF2E.3 (Optical Society of America, 2017).

[43] Helt LG, Yang Z, Liscidini M, Sipe JE (2010). Spontaneous four-wave mixing in microring resonators. Opt. Lett..

[44] Vernon Z (2017). Truly unentangled photon pairs without spectral filtering. Opt. Lett..

[45] Christensen, J. B., Koefoed, J. G., Rottwitt, K. & McKinstrie, C. J. Engineering spectrally unentangled photon pairs from nonlinear microring resonators through pump manipulation. Preprint at https://arxiv.org/abs/1711.02401 (2017). 10.1364/OL.43.000859 29444012

[46] Roslund J, de Araújo RM, Jiang S, Fabre C, Treps N (2014). Wavelength-multiplexed quantum networks with ultrafast frequency combs. Nat. Photonics.

[47] Humphreys PC (2014). Continuous-variable quantum computing in optical time-frequency modes using quantum memories. Phys. Rev. Lett..

[48] Sinclair N (2014). Spectral multiplexing for scalable quantum photonics using an atomic frequency comb quantum memory and feed-forward control. Phys. Rev. Lett..

[49] Lukens JM, Lougovski P (2017). Frequency-encoded photonic qubits for scalable quantum information processing. Optica.

[50] Grimau Puigibert M (2017). Heralded single photons based on spectral multiplexing and feed-forward control. Phys. Rev. Lett..

[51] Azuma K, Tamaki K, Lo HK (2015). All-photonic quantum repeaters. Nat. Commun..

[52] Agha I, Davanço M, Thurston B, Srinivasan K (2012). Low-noise chip-based frequency conversion by four-wave-mixing Bragg scattering in SiNx waveguides. Opt. Lett..

