# Supplementary material for: Frequency multiplexing for quasi-deterministic heralded single-photon sources
Source: Nat Commun. 2018 Feb 27;9:847. doi: 10.1038/s41467-018-03254-4 (PMC5829139; doi:10.1038/s41467-018-03254-4)
Supplement: Supplementary file 1 — Supplementary Information [file 41467_2018_3254_MOESM1_ESM.pdf]

Supplementary Note 1

Bragg scattering four-wave mixing

Bragg scattering four-wave mixing (BS-FWM) is a parametric process driven by two strong pump fields at ω_{P1} and ω_{P2} separated by $\Delta\omega = \omega_{\mathrm{P1}} - \omega_{\mathrm{P2}}$ that can convert an input field ω_{i} to $\omega_{\mathrm{t}} = \omega_{\mathrm{i}} - \Delta\omega$ (see Supplementary Figure 1a). The efficiency of the conversion is $\eta_{\mathrm{BS}} = \frac{k^2}{k^2+\kappa^2}\sin(\sqrt{k^2+\kappa^2}L)$ where $\kappa = 2P\gamma$ is the nonlinear strength, $k = \frac{1}{2}(\beta_{\mathrm{P1}} - \beta_{\mathrm{P2}} + \beta_{\mathrm{t}} - \beta_{\mathrm{i}})$ is the phase mismatch, L the interaction length, P is the power in each pump, γ is the nonlinear coefficient of the medium, and $\beta_{\mathrm{P1,P2,t,i}}$ the propagation vector for each of the participating fields. At the end of the interaction, the signal intensity is depleted by a factor of $1 - \eta_{\mathrm{BS}}$. For a perfectly phase-matched process ($k = 0$), complete conversion is achieved when the interaction strength $2\gamma PL$ equals $\pi/2$ (Supplementary Figure 1b). Tunable frequency conversion can be achieved by tuning the separation $\Delta\omega$ between the two strong pumps. Phase matching is achieved by symmetric placement of the pumps and the input and target fields around the zero-dispersion wavelength ($\beta^{(2)} = 0$). The acceptance bandwidth $\Delta\nu_{\mathrm{BS}}$ of this process is determined by higher order dispersion.

The setup used for this experiment is similar to [1, 2]. We use a 100-m long dispersion shifted fiber with the zero dispersion wavelength $\lambda_{\mathrm{zgvd}} = 1405$ nm as the nonlinear medium. The fiber is cooled using liquid nitrogen in order to remove spontaneous Raman noise. Pumps are generated by temperature stabilized distributed feed-back laser diodes and combined together using DWDMs. Within the bandwidth of the filter on the target channel, we measure a background of 3×10^{-3} photons per pump pulse duration. We achieve 93% conversion efficiency with this setup, as shown in Supplementary Figure 1c. The conversion efficiency is limited by the fluctuations in the pump power due to random triggering set by the CW nature of the photon source. With a fixed periodic pump trigger, we measure a conversion efficiency of 95%. Our filtering bandwidth at the target output is set to 100 GHz (Supplementary Figure 1e), which is less than the acceptance bandwidth of the process ($\Delta\nu_{\mathrm{BS}} = 190$ GHz).

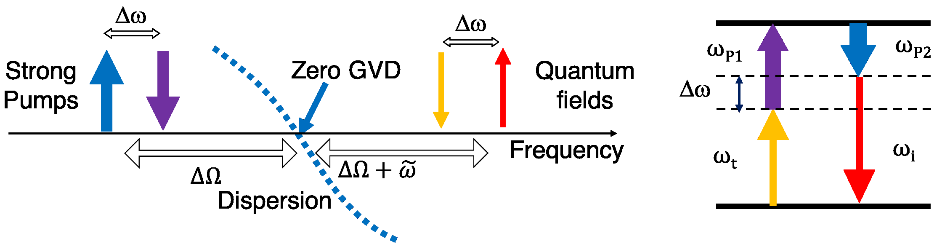

(a)

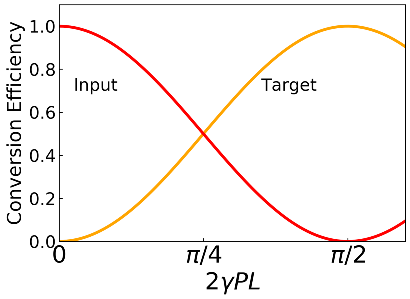

(b)

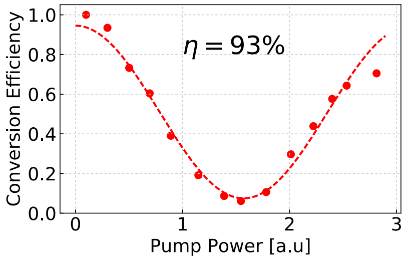

(c)

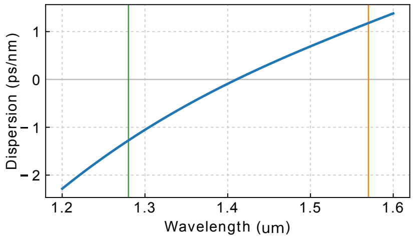

(d)

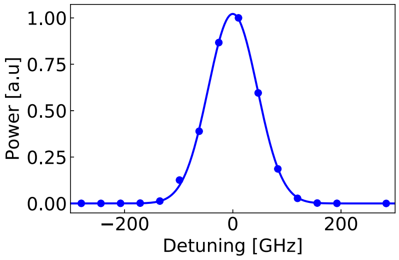

(e)

Supplementary Figure 1. **a)** Bragg scattering four-wave mixing (BS-FWM): Two strong pumps ω_{P1} and ω_{P2} drive the interaction between two fields ω_{s} and ω_{i}, where the separation $\Delta\omega = \omega_{\mathrm{P1}} - \omega_{\mathrm{P2}}$ determines the frequency shift between the signal and idler fields. Phase matching is achieved by symmetric placement of the pumps, and the signal, idler fields about the zero group velocity dispersion (zero GVD) wavelength of the interaction medium. **b)** BS-FWM conversion efficiency as function of interaction strength $2\gamma PL$, assuming perfect phase matching. Complete conversion is achieved when the interaction strength equals $\pi/2$. **c)** Measured conversion efficiency via depletion of the input. The conversion efficiency is measured to be 93%, limited by fluctuations in the pump power. **d)** Dispersion profile of the dispersion shifted fiber used in our implementation of BS-FWM. **e)** Measured bandwidth of the filtering setup centered at the target frequency.

Supplementary Note 2

Theory of multiplexed sources

We adapt the analysis presented in References [3, 4]. We assume that the SPDC source generates a two-mode squeezed state:

$$|\psi\rangle = \sqrt{1-|\xi|^2}\sum_{\mathrm{n=0}}^{n=\infty} \xi^n |n_{\mathrm{s}}, n_{\mathrm{i}}\rangle \tag{1}$$

where ξ is the squeezing parameter.

Assuming the use of bucket (non-photon number resolving detectors), the total heralding probability is obtained by summing over the probability of an n-photon fock state registering a 'click'on the detector, given by $[1-(1-\eta_{\mathrm{h}})^n]$, over all n.

$$p_{\mathrm{h}} = \frac{\eta_{\mathrm{h}}|\xi|^2}{1-(1-\eta_{\mathrm{h}})|\xi|^2} \tag{2}$$

where η_{h} is the net effciency on the heralding arm.
The conditional probability of detecting a single photon on the heralded arm is then given by:

$$p_{\mathrm{s}} = \frac{1}{p_{\mathrm{h}}}\eta_{\mathrm{d}}\eta_{\mathrm{h}}|\xi|^2(1-|\xi|^2) \tag{3}$$

where η_{d} is the net efficiency on the heralded photon arm. For N multiplexed sources, the probability that a heralding photon is registered in at least one of the N sources is given as:

$$p_{\mathrm{h}}^{\mathrm{mux}}(N) = 1-(1-p_{\mathrm{h}})^N \tag{4}$$

If the net efficiency of the switching network is η_{switch}, the corresponding total probability that a single photon is heralded at the ouput is given by combining Supplementary Equations 3 and 4:

$$p_{\mathrm{s}}^{\mathrm{mux}}(N) = \eta_{\mathrm{switch}} \times p_{\mathrm{s}} \times p_{\mathrm{h}}^{\mathrm{mux}}(N) \tag{5}$$

Here the loss factor η_{switch} for the fixed loss and log tree architectures is given as:

$$\eta_{\mathrm{switch}}^{\mathrm{fixed-loss}} = \eta_{\mathrm{s}} \tag{6}$$

$$\eta_{\mathrm{switch}}^{\mathrm{log-tree}} = \eta_{\mathrm{s}}^{\lceil \log_2 N \rceil} \tag{7}$$

where η_{s} is the switching effciency per switch. The expressions for the multi-pass scheme are slightly more involved. We assume optimization similar to that in Reference [5] where losses are minimized by routing photons from the the last heralded slot in case of temporal multiplexing. The total heralding probability in this case is given by:

$$p_{\mathrm{s}}^{\mathrm{mux}} = \sum_{\mathrm{j=1}}^{N} (1-p_{\mathrm{h}})^{N-j} p_{\mathrm{h}} \times p_{\mathrm{s}} \eta_{\mathrm{s}}^{N-j} \tag{8}$$

In Supplementary Equation 8 the first term is the probability that no photon was heralded in the last $N-j$ slots, and the corresponding switching losses are equal to η_{s}^{N-j}. We note that we do not consider the cases where multi-photon emission is detected as a single photon event due to switching losses. In order to isolate effects of switching losses, we assume all other components including detection to be perfect ($\eta_{\mathrm{d}} = 1, \eta_{\mathrm{h}} = 1$).

While determining the scaling performance of the multiplexed sources for various N, we optimize the mean photon number (or equivalently the squeezing parameter) for each N. The squeezing parameter ξ is related to the mean photon number in the signal and idler modes as:

$$\mu = \frac{|\xi|^2}{1-|\xi|^2} \tag{9}$$

For small heralding probability p_{h}, the probability that atleast one multiplexed source triggers is Np_{h}. As N increases, optimal performance is achieved for lower mean photon number μ, as shown in Supplementary Figure 2. Therefore, to obtain the scaling performance of the schemes we optimize μ for each N, maintaining this μ across schemes.

Finally, the conditional multi-photon probability for a given squeezing parameter is (ignoring switching losses):

$$\begin{aligned} p_{\mathrm{multi}} &= |\xi|^4 \\ &= \left(\frac{\mu}{1+\mu}\right)^2 \end{aligned} \tag{10}$$

As the mean photon number is reduced for increasing N, the multi-photon noise correspondingly reduces for large N.

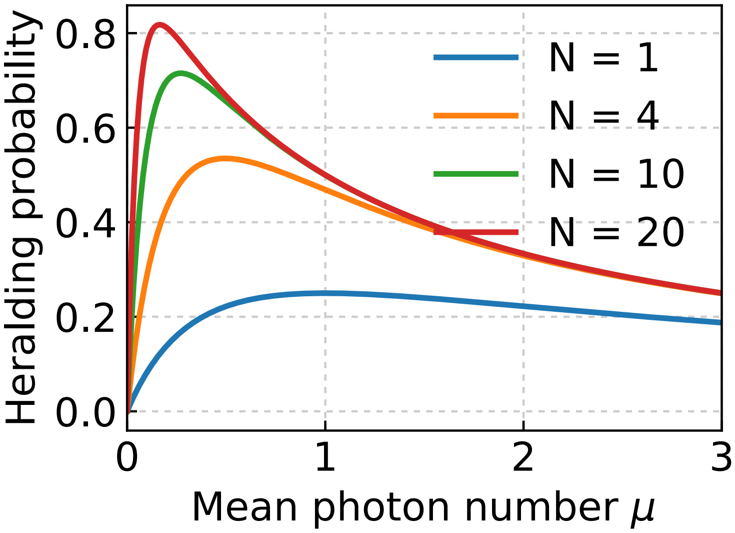

Supplementary Figure 2. Heralding probability for N multiplexed sources as a function of the mean photon number μ in the signal and idler modes, for a lossless switching network. Optimal μ reduces as N increases.

Supplementary Note 3

Source Characterization

We characterize the spectrum of our idler (heralding) photons using a single photon spectrometer (Ocean Optics). The results are shown in Supplementary Figure 3. The heralding photons are filtered into 100 GHz wide channels using reflecting Bragg gratings. The highlighted regions reflect the corresponding heralded channels.

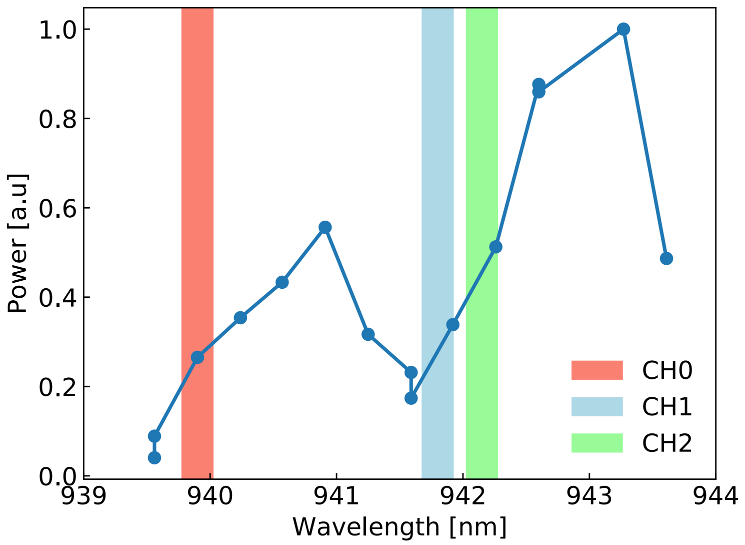

Supplementary Figure 3. Spectrum of the heralding photons measured using a single photon spectrometer. The heralding photons are filtered into 100 GHz wide channels using reflecting Bragg gratings. The highlighted regions reflect the corresponding heralded channels: CH0, CH1, CH2.

Supplementary Note 4

Characterization of system efficiency

We measure the heralding efficiency on the multiplexed photon arm by measuring the ratio of the detected coincidences to the heralding rate, as shown in Supplementary Figure 4. The raw heralding efficiency is about 2.3%. Without the multiplexing setup in place, we measure a heralding efficiency of 3%, corresponding to a 1.3 dB loss due to the multiplexing BS-FWM setup. The losses in the path of the multiplexed photon after collection from the SPDC source were measured to be: 1.3 dB BS-FWM setup (WDMs and nonlinear fiber), 1 dB free-space filtering grating, 2.5 dB fiber-coupling after filtering and 3 dB detection loss. After accounting for detection loss, we infer a heralding probability of 4.6%. We estimate about 8 dB losses at collection from the SPDC source, primarily due to mode-mismatch and transmission loss from filtering optics.

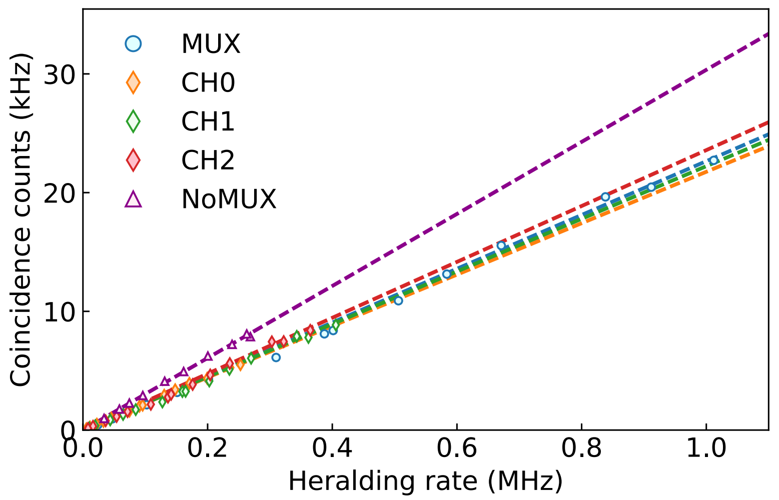

Supplementary Figure 4. Characterization of heralding efficiency. The efficiency is given by the slope of the heralding rate vs heralded photon rate (measured as coincidence counts). We measure a heralding efficiency of 2.3% with the multiplexing setup in place and an efficiency of 3% without. This corresponds with the estimated 1.3 dB transmission loss through the BS-FWM setup measured using a classical input.

Supplementary Note 5

Comparison with previous demonstrations of multiplexed photon sources

In Supplementary Table 1, we compare the the performance characteristics of our frequency multiplexed source with previous demonstrations of multiplexing using different schemes and platforms. Generated rates are calculated after accounting for detector inefficiency. We note that the maximum switching speed of our system is only limited by the amplification required for the BS-FWM pumps. In principle, our system can be operated at a repetition rate of $1/\Delta\nu_{\mathrm{BS}}$ where $\Delta\nu_{\mathrm{BS}}$ is the acceptance bandwidth of BS-FWM, which is 100 GHz for our current implementation.

Reference	Multiplexing scheme	Platform	#Modes N	Single-photon detection rate (kHz)	Enhancement factor	$g^{(2)}(0)$	Heralding efficiency	Max. switching speed
This work	Frequency	Fiber	3	23 (46†)	2.2	0.07	2.3% (4.6%†)	5 MHz
Kaneda et al. [5]	Time	Free space	30	11 (19.3†)	6	0.48	22% (38.6%†)	50 kHz
Xiong et al. [6]	Time	Fiber	4	0.6	2	...	...	...
Mendoza et al. [7]	Space-Time	Fiber	8	0.4	1.8	...	$\leq 0.5\%^*$	500 kHz
Puigibert et al.[8]	Frequency	Fiber	3	0.4	1	0.06	...	...
Francis-Jones et al.[9]	Space	Fiber	2	0.6	1.4	0.05	...	...
Ma et al.[10]	Space	Free space	4	0.7	1.4	0.1	...	15 MHz
Collins et al. [11]	Space	Integrated	2	0.02	1.6	0.2	1%	1 MHz

†generation rates are estimated after correcting for detection loss

*estimated from reported data

Supplementary Table 1. Comparison of the performance of the frequency multiplexed source with other demonstrations of multiplexed single photon sources using various schemes and platforms.

Supplementary Note 6

Frequency multiplexing scaling

Here we show how an implementation of frequency multiplexing using BS-FWM can support large number of frequency channels without drop in conversion efficiency and achieve a 50% single-photon heralding probability with just 10 multiplexed modes.

We introduce for convenience average frequency $\Delta\Omega = (\omega_{\mathrm{P1}} + \omega_{\mathrm{P2}})/2 - \omega_{\mathrm{ZDW}}$ and of the frequency offset $\widetilde{\omega} = \omega_{\mathrm{i}} - (\Delta\omega/2 + \Delta\Omega + \omega_{\mathrm{ZDW}})$, where $\Delta\omega = \omega_{\mathrm{P1}} - \omega_{\mathrm{P2}}$ is the separation between the pumps (see Supplementary Figure 1a). The phase mismatch $k = \beta(\omega_{\mathrm{t}}) - \beta(\omega_{\mathrm{i}}) + \beta(\omega_{\mathrm{P1}}) - \beta(\omega_{\mathrm{P2}})$ can then be expanded around the zero dispersion frequency ω_{ZDW} as follows:

$$k = \frac{\beta^{(3)}}{6}\left[\left(\Delta\Omega + \tilde{\omega} - \frac{\Delta\omega}{2}\right)^3 - \left(\Delta\Omega + \tilde{\omega} + \frac{\Delta\omega}{2}\right)^3 - \left(-\Delta\Omega - \frac{\Delta\omega}{2}\right)^3 + \left(-\Delta\Omega + \frac{\Delta\omega}{2}\right)^3\right] + \mathcal{O}(\beta^{(4)})$$

$$k = \frac{\beta^{(3)}}{6}\left[3\tilde{\omega}\Delta\omega(\tilde{\omega} + 2\Delta\Omega)\right] \qquad (11)$$

This shows that shows that phase-matching can always be fulfilled by choosing $\widetilde{\omega} = 0$. While the condition $k = 0$ is always satisfied, for large detuning $\Delta\omega$ between the input and the target frequency ω_{t}, the acceptance bandwidth is modified and depends on the detuning $\Delta\omega$. The target bandwidth must match the acceptance bandwidth for optimal conversion. As shown in Figure 5 of the main text, the acceptance bandwidth reduces by a factor of 2 (from 190 GHz to 90 GHz) when $\Delta\omega$ is tuned over 1 THz.

Supplementary Figure 5 shows the calculated scaling performance of the frequency multiplexed source for 10 modes. We assume a feasible value for detector and fiber-collection efficiency of 90%. Heralding efficiencies as high as 50% can be achieved using just 10 multiplexed modes.

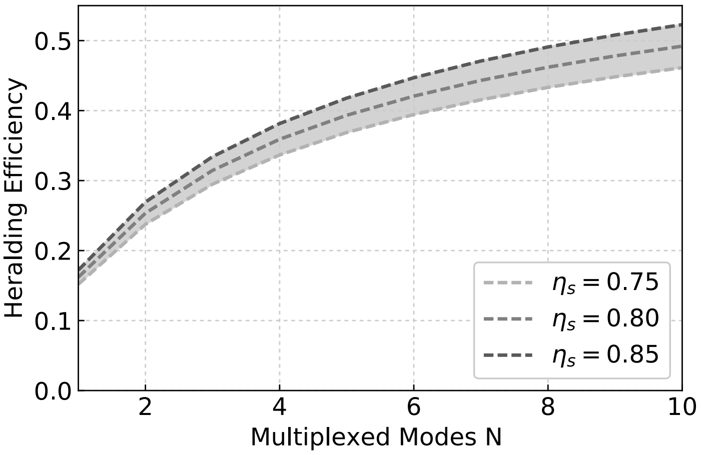

Supplementary Figure 5. Scaling performance of frequency multiplexing for 10 frequency modes, assuming a combined detection and fiber-collection efficiency of 90%, for varying multiplexing system efficiencies ($\eta_\mathrm{s} = 0.75, 0.80, 0.85$). Single-photon heralding efficiencies as high as 50% can be achieved with just 10 frequency modes.

Supplementary Note 7

Generation of indistinguishable photons using SPDC

In order to generate indistinguishable photons using frequency multiplexing, the correlations between the heralding and the heralded photon must vanish within the filtering bandwidth of each channel (set less than or equal the BS-FWM bandwidth). Currently, we use a 1 cm PPLN crystal with Type 0 phase matching. Due to the highly non-degenerate nature of our downconversion source, the resulting phase matching condition is tight. This results in highly correlated photon pairs within the filtering bandwidth. However, using a shorter PPLN crystal to reduce the interaction length relaxes this condition sufficiently to allow the generation of spectrally pure photons after filtering. In Supplementary Figure 6, we show that using a short 1 mm PPLN crystal and Type 0 phase matching results in a joint spectral intensity (JSI) after filtering (bandwidth - 100 GHz) with a Schmidt number $K = 1.12$. The expected indistinguishability is $1/K = 0.89$.

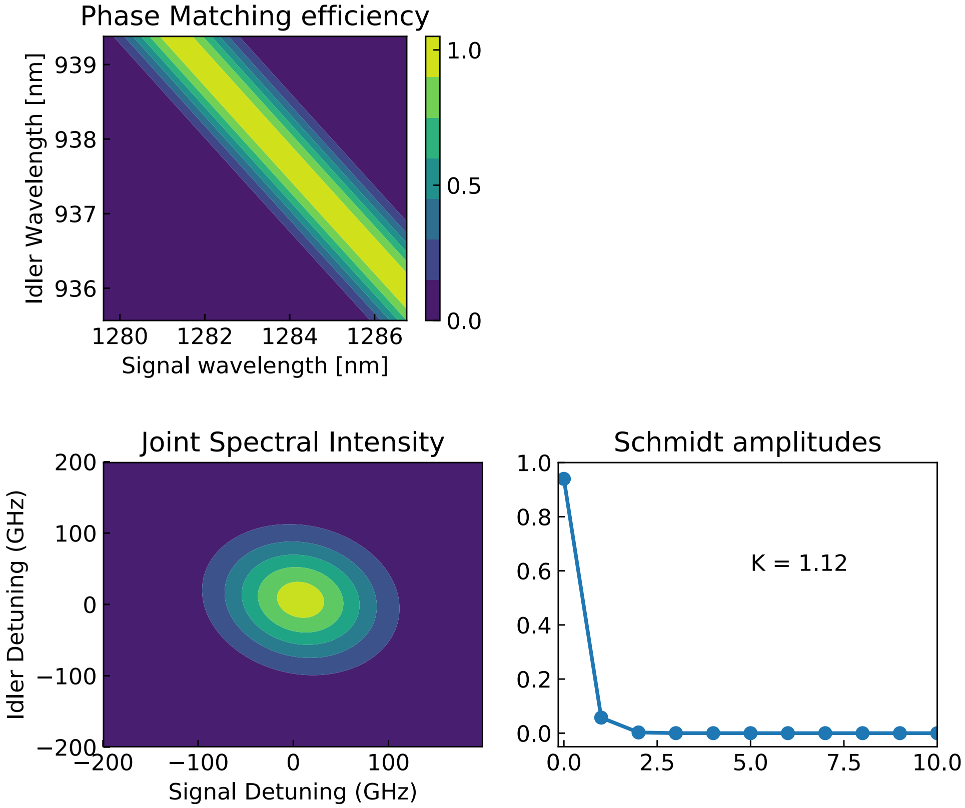

Supplementary Figure 6. Calculation of phase matching efficiency, joint spectral intensity after filtering(JSI) and Schmidt amplitudes for a 1 mm PPLN crystal pumped with a 3 nm broad pulsed source. The JSI is calculated assuming a filtering bandwidth of 100 GHz, resulting in a Schmidt number $K = 1.12$.

SUPPLEMENTARY REFERENCES

[1] Farsi, A., Clemmen, S., Ramelow, S. & Gaeta, A. L. Low-Noise Quantum Frequency Translation of Single Photons. In *Conference on Lasers and Electro-Optics*, FM3A.4 (Optical Society of America, 2015).

[2] Clemmen, S., Farsi, A., Ramelow, S. & Gaeta, A. L. Ramsey interference with single photons. *Phys. Rev. Lett.* **117**, 223601 (2016).

[3] Christ, A. & Silberhorn, C. Limits on the deterministic creation of pure single-photon states using parametric down-conversion. *Phys. Rev. A* **85**, 023829 (2012).

[4] Bonneau, D., Mendoza, G. J., O'Brien, J. L. & Thompson, M. G. Effect of loss on multiplexed single-photon sources. *New J. Phys.* **17**, 043057 (2015).

[5] Kaneda, F. *et al.* Time-multiplexed heralded single-photon source. *Optica* **2**, 1010–1013 (2015).

[6] Xiong, C. *et al.* Active temporal multiplexing of indistinguishable heralded single photons. *Nat. Commun.* **7**, 10853 (2016).

[7] Mendoza, G. J. *et al.* Active temporal and spatial multiplexing of photons. *Optica* **3**, 127 (2016).

[8] Grimau Puigibert, M. *et al.* Heralded Single Photons Based on Spectral Multiplexing and Feed-Forward Control. *Phys. Rev. Lett.* **119**, 083601 (2017).

[9] Francis-Jones, R. J. A., Hoggarth, R. A. & Mosley, P. J. All-fiber multiplexed source of high-purity single photons. *Optica* **3**, 1270–1273 (2016).

[10] Ma, X.-s., Zotter, S., Kofler, J., Jennewein, T. & Zeilinger, A. Experimental generation of single photons via active multiplexing. *Phys. Rev. A* **83**, 043814 (2011).

[11] Collins, M. J. *et al.* Integrated spatial multiplexing of heralded single-photon sources. *Nat. Commun.* **4**, 2582 (2013).
